# ST18 Enhances PV-IgG-Induced Loss of Keratinocyte Cohesion in Parallel to Increased ERK Activation

**DOI:** 10.3389/fimmu.2019.00770

**Published:** 2019-04-17

**Authors:** Mariya Y. Radeva, Elias Walter, Ramona Alexandra Stach, Amir S. Yazdi, Nicolas Schlegel, Ofer Sarig, Eli Sprecher, Jens Waschke

**Affiliations:** ^1^Department I, Institute of Anatomy and Cell Biology, Ludwig-Maximilians-Universität (LMU) Munich, Munich, Germany; ^2^Department of Dermatology, University Medical Center Tübingen, Eberhard Karls University, Tübingen, Germany; ^3^Department of Dermatology, RWTH Aachen, Aachen, Germany; ^4^Department of General, Visceral, Vascular and Paediatric Surgery, Julius-Maximilians-Universität, Würzburg, Germany; ^5^Department of Dermatology, Tel-Aviv Sourasky Medical Center, Tel-Aviv, Israel; ^6^Department of Human Molecular Genetics and Biochemistry, Sackler Faculty of Medicine, Tel-Aviv University, Tel-Aviv, Israel

**Keywords:** pemphigus, desmosome, desmoglein, ST18, ERK, cytokines

## Abstract

Pemphigus is an autoimmune blistering disease targeting the desmosomal proteins desmoglein (Dsg) 1 and Dsg3. Recently, a genetic variant of the Suppression of tumorigenicity 18 (ST18) promoter was reported to cause ST18 up-regulation, associated with pemphigus vulgaris (PV)-IgG-mediated increase in cytokine secretion and more prominent loss of keratinocyte cohesion. Here we tested the effects of PV-IgG and the pathogenic pemphigus mouse anti-Dsg3 antibody AK23 on cytokine secretion and ERK activity in human keratinocytes dependent on ST18 expression. Without ST18 overexpression, both PV-IgG and AK23 induced loss of keratinocyte cohesion which was accompanied by prominent fragmentation of Dsg3 immunostaining along cell borders. In contrast, release of pro-inflammatory cytokines such as IL-1α, IL-6, TNFα, and IFN-γ was not altered significantly in both HaCaT and primary NHEK cells. These experiments indicate that cytokine expression is not strictly required for loss of keratinocyte cohesion. Upon ST18 overexpression, fragmentation of cell monolayers increased significantly in response to autoantibody incubation. Furthermore, production of IL-1α and IL-6 was enhanced in some experiments but not in others whereas release of TNF-α dropped significantly upon PV-IgG application in both EV- and ST18-transfected HaCaT cells. Additionally, in NHEK, application of PV-IgG but not of AK23 significantly increased ERK activity. In contrast, ST18 overexpression in HaCaT cells augmented ERK activation in response to both c-IgG and AK23 but not PV-IgG. Because inhibition of ERK by U0126 abolished PV-IgG- and AK23-induced loss of cell cohesion in ST18-expressing cells, we conclude that autoantibody-induced ERK activation was relevant in this scenario. In summary, similar to the situation in PV patients carrying ST18 polymorphism, overexpression of ST18 enhanced keratinocyte susceptibility to autoantibody-induced loss of cell adhesion, which may be caused in part by enhanced ERK signaling.

## Introduction

Pemphigus vulgaris (PV) is a potentially fatal autoimmune disease of the skin and the mucous membranes. The skin disorder is manifested by mucocutaneous blister formation caused by loss of keratinocytes intercellular cell adhesion, which is primarily promoted by IgG autoantibodies directed against desmosomal adhesion molecules such as desmoglein (Dsg) 1 and Dsg 3 (1). However, autoantibodies targeting desmocollin (Dsc) 3, which are rarely present in cases when antibodies against Dsg3 are missing, were also shown to be pathogenic (2–4). These desmosomal cadherins are transmembrane proteins forming the adhesive core of desmosomes, a special intercellular junction maintaining the mechanical integrity of tissues and bearing tension primarily if exposed to high levels of mechanical stress such as in the epidermis (5). With their cytoplasmic tail, via Plakoglobin (PG), and Desmoplakin (DP), the cadherins are linked to the intermediate filament skeleton, another structural unit providing mechanical strength to cells (6, 7). Besides their mechanical functions, desmosomes serve as signaling hubs coordinating tissue-specific functions with intercellular adhesion, which is required for instance during wound healing (8).

In response to binding of pemphigus autoantibodies to the keratinocyte surface, Dsg3-mediated homophilic interaction is impaired and desmosomal proteins are depleted from the membrane leading to keratin retraction and Dsg internalization (9–12). These structural alterations are induced by various PV-mediated outside-in intracellular signaling events such as p38 mitogen-activated protein kinase (p38MAPK) (13–15) and extracellular-signal regulated kinase (ERK) (16, 17). Signaling mechanisms have been shown to be important for loss of cell adhesion (17) and the importance of several signaling mechanisms is highlighted by the protective effects of pharmacological inhibitors *in vivo, in vitro*, or *ex vivo* (18–20).

Despite all efforts, the etiology of the disease is still not completely understood. Interestingly studies reporting ethnic susceptibility and familial occurrence of the PV- skin disorder provided evidence for a genetic predisposition to PV (21–25). In this respect, most of the reports provided data for PV associated genes belonging to a human leukocyte antigen (HLA) locus. Few studies, however, described PV genetic association of non-HLA genes (25). Amongst the latter is suppression of tumorigenicity 18 (ST18), the product of which functions as a transcription factor and thereby controls the mRNA levels of numerous proappoptotic and pro-inflammatory genes (26), participating in regulation of processes with potential relevance for loss of cell adhesion in PV (27, 28). Recently, a genetic variant located within the ST18 promoter was reported to cause ST18 up-regulation, associated with PV-IgG-mediated increase in cytokine secretion and more prominent loss of keratinocytes cohesion (29). In line with this, single nucleotide polymorphisms (SNP) identified in the ST18 gene were proposed to predispose to PV in a population-specific manner. SNPs promoted augmentation in ST18 expression was shown to be associated with more severe disease manifestation, indicative for the direct role of ST18 in PV pathogenesis (30, 31).

In the present study we investigated in both HaCaT and normal human epidermal keratinocytes (NHEK) the release of key pro-inflammatory molecules such as IL-1α, IL-6, TNF-α, and IFN-γ upon PV-IgG treatment. Additionally, the effect of ST18 overexpression on cytokine release but also on the modulation of pemphigus-associated ERK signaling was evaluated since both events would render keratinocytes more susceptible to PV-IgG-induced loss of keratinocyte adhesion.

## Materials and Methods

### Cell Culture, Isolation of Human Primary Keratinocytes

For all experiments, primary normal human epithelial keratinocytes (NHEK) and HaCaT, a spontaneously immortalized human skin keratinocyte cell line were used. HaCaT cells were maintained in Dulbecco's Modified Eagle Medium (DMEM), (Life Technologies; Carlsbad; CA; USA) supplemented with 10% FCS (Biochrom, Berlin, Germany), 50 U/ml penicillin and 50 U/ml streptomycin, both antibiotics were purchased from AppliChem, Darmstadt, Germany.

NHEK were generated at Universitäts-Hautklinik Tübingen. The procedure was approved by the medical ethical committee of the Eberhard Karls University Tübingen (ethical approval: 547/2011BO2). Briefly, the cells were isolated from juvenile foreskin derived from patients, who have given written informed consent. The skin was divided into epidermis and dermis by using 50 mg/ml Dispase II (Roche; Basel, Switzerland). Epidermal cells were then separated from each other by using a trypsin-EDTA solution (Merck, Darmstadt, Germany) and afterwards cultivated in CnT-07 medium (CELLnTEC Advanced Cell Systems AG, Switzerland) containing 10 μg/ml gentamycin and 0.25 μg/ml amphotericin B. The cells were cultured in low calcium conditions (0.06 mM Ca^2+^). 24 h prior treatment, cell differentiation was induced by increasing the Ca^2+^ concentration to 1.8 mM. All experiments were conducted on NHEK cells between passages 3 to 8.

Both cell lines were grown in a humidified atmosphere containing 5% CO_2_ at 37°C. After cells reached confluency the medium was changed and 24 h later cell monolayers were treated accordingly.

### Constructs and Transfection

The human ST18 construct (pCMV6-ST18-Myc/DDK) was generated by inserting the translated region of a human ST18 cDNA into the pCMV6-Entry target vector (Origene Technologies Company, Rockville, MD, USA). The latter was used also for a control transfection.

ST18 overexpression was accomplished by transient transfection with a plasmid DNA into HaCaT cells at 80% confluence using Turbofect transfection reagent (Fermentas, ThermoFisher Scientific). 24 h after transfection, medium was changed and cell monolayers were further exposed to treatment with various IgG fractions.

### RNA Isolation, Complementary DNA (cDNA) Preparation, and PCR Performance

To confirm sufficient transfection efficiency with ST18 construct, PCR was performed with cDNA transcribed from an equal amount of total RNA. The latter was isolated by using RNeasy Plus mini kit (Qiagen, Venlo, Netherlands). cDNA was synthesized through the usage of SuperScript II reverse transcriptase kit (Invitrogen, ThermoFisher Scientific). All preparations were carried out according to manufacturer's instructions.

The transfection efficiency of each experiment requesting ST18 overexpression was tested by PCR using following primer pair:

hST18-164bp-Fw CACTAATCCAGGAGCTCAGTGTTG

hST18-164bp-Rev CCTGTCACTGCGGTCTTCTTG

To validate the PCR assay, positive (pCMV-ST18-Myc/DDK vector) and negative (instead of cDNA, H_2_O was used as a template) controls were used. An empty negative control reflects the lack of contaminations, whereas manifestation of amplicon with correct size in the positive control guarantees accurate PCR conditions.

PCR was carried out as follows: 95°C for 3 min, followed by 22 cycles at 95°C for 30 s, 60°C for 30 s and 72°C for 30 s, the final extension was at 72°C for 5 min. Then, the PCR products were visualized by gel electrophoresis.

### Dispase-Based Keratinocytes Dissociation Assay

Keratinocytes were seeded in 24-well plates and grown to confluency. NHEK cells were subsequently differentiated for 24 h by increasing the Ca^2+^-concentration to 1.8 mM. HaCaT monolayers were transiently transfected either with control or pCMV-ST18-Myc/DDK vector 24 h prior to experimental procedure. Monolayers treated with PBS (Vehicle) or control IgG (c-IgG) for 24 h were considered as controls. Experimental conditions were treated with AK23 or different PV-IgG in a similar fashion.

The loss of cell cohesion was tested by dispase-based dissociation assay. The latter was performed as described elsewhere (32, 33). Briefly, after treatment, cell monolayers were washed with pre-warmed Hank's Balanced Salt Solution (HBSS) and subsequently released from the well-bottom by incubation with 2.4 U/ml Dispase II (Sigma-Aldrich) dissolved in HBSS for 20 min at 37°C. Next, the protease solution was substituted by HBSS and each condition was treated additionally for 10 min with thiazolyl blue tetrazolium bromide (MTT) (Sigma-Aldrich) at a final concentration of 10 μM to better visualize the monolayer sheets. The integrity of a single intact monolayer was compromised by applying controlled shear stress through pipetting the monolayers with an electrical 1 ml pipette. Resulting fragments were counted under a binocular microscope (Leica, Mannheim, Germany) as their number is an inverse measure of intercellular adhesion. Higher number of fragments correspond to stronger loss of intercellular adhesion. A Canon EOS 750D camera was used to record the dissociation experiments. Each experiment was repeated 6 to 10 times.

### Analysis of the Cytokine Secretion

Simultaneous quantitative cytokine measurements were accomplished by high-throughput bead-based multiplex assays. A Milliplex kit (HCYTOMAG-60K, Merck KGaA, Darmstadt, Germany), precustomized for cytokines of interest, i.e., IL-1α, IL-6, IFN-γ, TNF-α, was used. Along with the serial standards, each sample was analyzed in duplicate or triplicate following the manufacturer's instructions. Collection of the data was achieved by using Luminex LX100/200 system (Luminex Corporation, Austin, Texas). Data analysis was performed using MILLIPLEX® Analyst 5.1.Software (Merck KGaA, Darmstadt, Germany). To estimate the cytokine concentration (expressed in pg/ml), standard curves derived from known reference concentrations supplied by the manufacturing company were utilized.

### Immunostaining

Human keratinocytes (HaCaT and NHEK) were grown to confluence on glass coverslips. Twenty four hours prior treatment, the medium was changed and in the case of NHEK cells, the Ca^2+^ concentration was increased to 1.8 mM in order to induce cell differentiation. After 24 h of incubation at 37°C with different IgG fractions the culture medium was removed, cell monolayers were washed with PBS and subsequently fixed (with 2% paraformaldehyde (PFA) for 10 min) and permeabilized (with 0,1% Triton-X-100 for 5 min). To prevent unspecific binding the intact monolayers were blocked with a mix of 1% normal goat serum and 3% bovine serum albumin solved in PBS for at least 30 min at room temperature (RT). Consequent overnight incubation at 4°C with primary rabbit anti-Dsg3 antibody (Biozol Diagnostica Vertrieb GmbH, Germany) was performed. After washing with PBS, the cell monolayers were incubated with Cy3- conjugated secondary goat-anti-rabbit antibody (Dianova, Hamburg, Germany) for an hour at RT. After rinsing the monolayers with PBS and H_2_O the cells were mounted on a glass slide with N-propyl gallate, an anti-fade reagent, which is used to reduce photobleaching of the fluorescent probes. Images were collected with 63xNA1.4 PL APO objective using a Leica SP5 confocal microscope.

### PV Sera and Purification

Patient blood sera were obtained from the Department of Dermatology and Allergology, Philipps-Universität Marburg, Marburg, Germany. Prior serum collection, all patients gave their written informed consent. For disease determination, clinical and histological studies were carried out and the antibody scores for aDsg1 and 3 were obtained during disease diagnosis. Control serum was donated from healthy volunteers. Before experimental usage, patient sera were purified by column affinity chromatography as described elsewhere (34). Briefly, Protein-A Agarose (ThermoFisher, Waltham, USA) was washed with PBS. Subsequently, beads were incubated with patient sera for 2 h at room temperature on a rotator, which enables IgG binding to the beads. After washing the beads with PBS, IgGs were detached from the Agarose by using sodium citrate (20 mM, pH 2.4) followed up by neutralization with sodium carbonate. Next, a filter unit (Amicon Ultra−4, 100 k; Merck Millipore, Darmstadt, Germany) was used to wash the resulting salts out of the IgG fractions by centrifugation at 19,000 g for 20 min. As a final step the IgGs were resuspended in PBS. Protein amount of purified IgG fractions was determined by Bicinchoninic acid kit (BCA) (Thermo Fisher Scientific, Schwerte, Germany) and used for further experiments at a dilution of 1:50.

The monoclonal antibody AK23 reacting to Dsg3 was purchased from Biozol (Eching, Germany).

### Triton X-100 Protein Fractionation, Electrophoresis, Western Blotting, and Tested Antibodies

Signaling pathway modulation was examined in either non-transfected or transfected keratinocytes exposed to different IgGs or AK23 for a time period of 30 min. After the incubation was accomplished, cell monolayers were washed with ice cold PBS. As next, extraction buffer (0.5% Triton X-100, 50 mmol/L MES, 25 mmol/L EGTA, 5 mmol/L MgCl_2_) containing 0.1% of Leupeptin, Pepstatin and Aprotinin as well as 1% PMSF was applied for 15 min, on ice, under moderate shaking. Lysates were collected by scraping. In order to achieve successful fractionation of the cytoskeletal bound insoluble from the soluble fraction, a centrifugation step at 19,000 g for 10 min at 4°C was performed. Resulting supernatants defined the cytosolic pools. The obtained pellet, representing the cytoskeletal bound fraction, was further washed with Triton extraction buffer, centrifuged for 10 min and consequently re-suspended in SDS-buffer (25 mmol/l HEPES, 2 mmol/l EDTA, 25 mmol/l NaF, and 1% SDS, pH 7,4, complete Protease Inhibitor Cocktail). After sonication, protein concentration of both fractions was determined by Pierce BCA Protein Assay Kit (Thermo Fisher Scientific, Schwerte, Germany), following the manufacturer's instructions. Equal amounts of samples were mixed with Laemmli buffer and consequently subjected to Western blotting as described elsewhere (17).

Following primary antibodies were incubated overnight at 4°C in 5% bovine serum albumin (BSA) in Tris-buffered-saline (TBS) with 0.05% tween (TSB-T): p-ERK (Cell Signaling Technologies, Cambridge, United Kingdom), p44/42 MAPK pAB (Erk1/2) (Cell Signaling Technologies), GAPDH mAB (Santa Cruz), Desmoplakin I/II pAB (H-300) (Santa Cruz). Following peroxidase coupled secondary antibodies were used for 1 h at room temperature in TBS-T: HRP-coupled goat anti-mouse, a HRP-coupled goat anti-rabbit (Dianova, Hamburg, Germany). Afterwards antibodies were visualized by chemiluminescence with ECL reaction (GE Healthcare, Munich, Germany).

### Data Analysis and Statistic

One-way ANOVA followed by Bonferroni correction (GraphPad Software, La Jolla, CA) was used to assess the differences among three or more treatments in dispase-based assay. To determine the cell response to different treatments in cells transfected with EV and ST18- vector either two-way ANOVA followed by Sidak's multiple comparisons test or multiple *t*-tests corrected by Holm-Sidak's method were applied as indicated. Each data point represents one single experiment. Data are presented as mean ± SEM. *P*-values less or equal to 0.05 assume significant differences between analyzed groups.

## Results

### Pathogenicity of Pemphigus Autoantibodies on Human Keratinocytes

**Figure 1:**
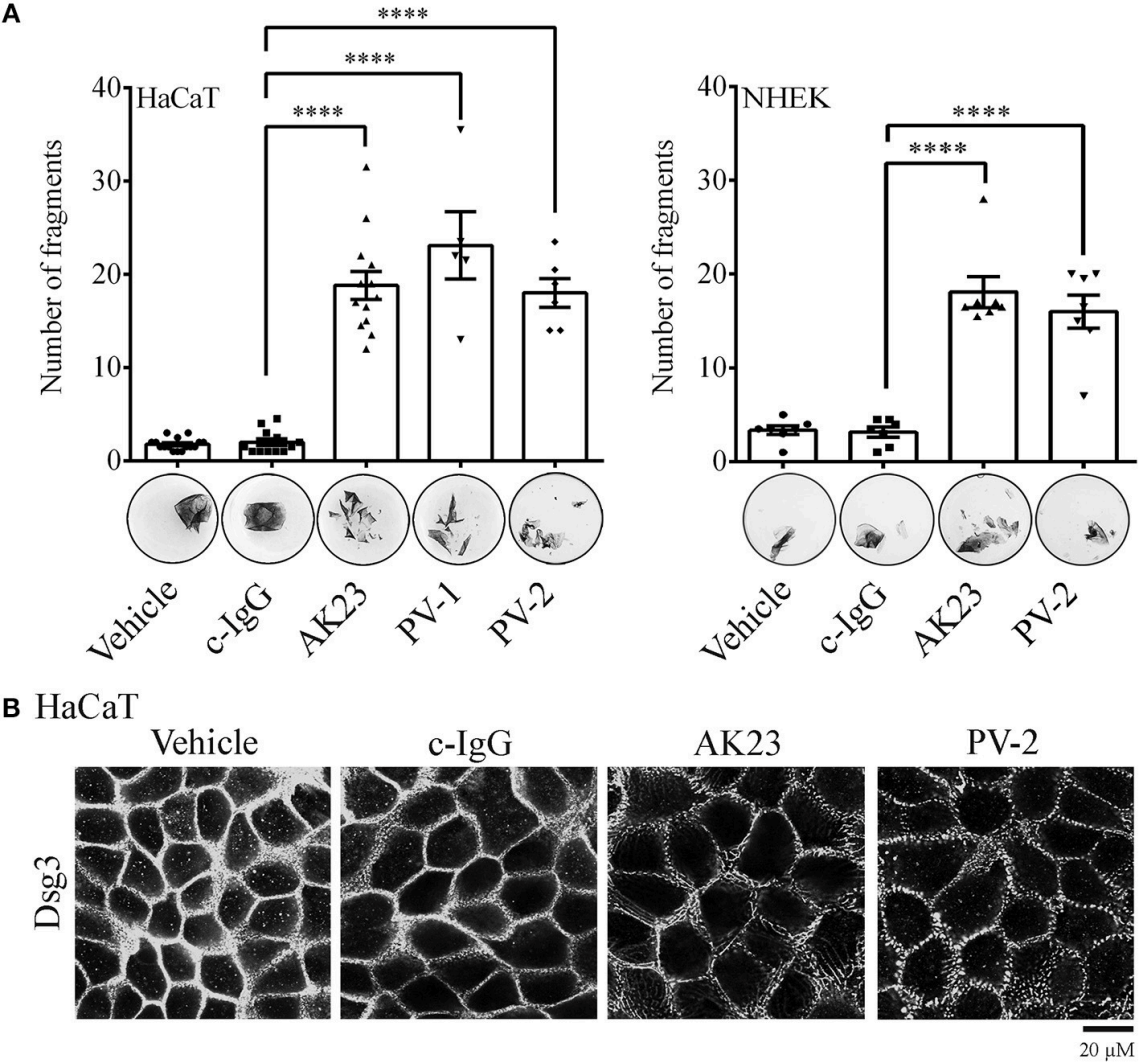
Pemphigus autoantibody application induces loss of keratinocyte cohesion and fragmentation of Dsg3 immunostaining. **(A)** Dispase-based dissociation assay in human HaCaT and NHEK keratinocytes after treatment with pemphigus autoantibodies. Besides PBS-treated monolayers (Vehicle), cells exposed to c-IgG were also used as a control. Representative images of cell sheets pre-stained with MTT and subjected to defined mechanical stress (*n* ≥ 6, One-way ANOVA, ****p ≤ 0.0001 vs. c-IgG). **(B)** Dsg3 immunostaining in response to pathogenic autoantibodies (PV-IgG and monoclonal autoantibody AK23, targeting Dsg3). Representative images of *n* ≥ 4 (Scale bar: 20 μm).

The pathogenic effect of IgG fractions derived either from an active pemphigus mouse model (AK23, a monoclonal autoantibody targeting Dsg3, (35)) or from pemphigus patients (PV-IgG) was evaluated by dispase-based dissociation assay in cultured immortalized HaCaT and primary normal human epidermal keratinocytes (NHEK) (Figure 1A). In contrast to control conditions, in which confluent monolayer exposed to PBS (vehicle) or control IgG (c-IgG) obtained from healthy volunteers remained intact, application of both AK23 and PV-IgG (PV-1 and PV-2) for 24 h induced loss of cell adhesion.

In HaCaT cell monolayers, the effect of pathogenic IgG-fractions on desmosomes was visualized by Dsg3 immunostaining. In all controls (vehicle and c-IgG), linearly organized Dsg3 staining at cell borders was observed. In contrast, 24 h incubation with autoantibodies induced fragmentation of the Dsg3 staining pattern along the borders (Figure 1B). Under the same conditions, NHEKs exhibited changes similar to those observed in HaCaT cells (Supplementary Figure 1).

### ST18 Overexpression Promotes PV-IgG-Mediated Loss of Keratinocyte Cohesion

**Figure 2:**
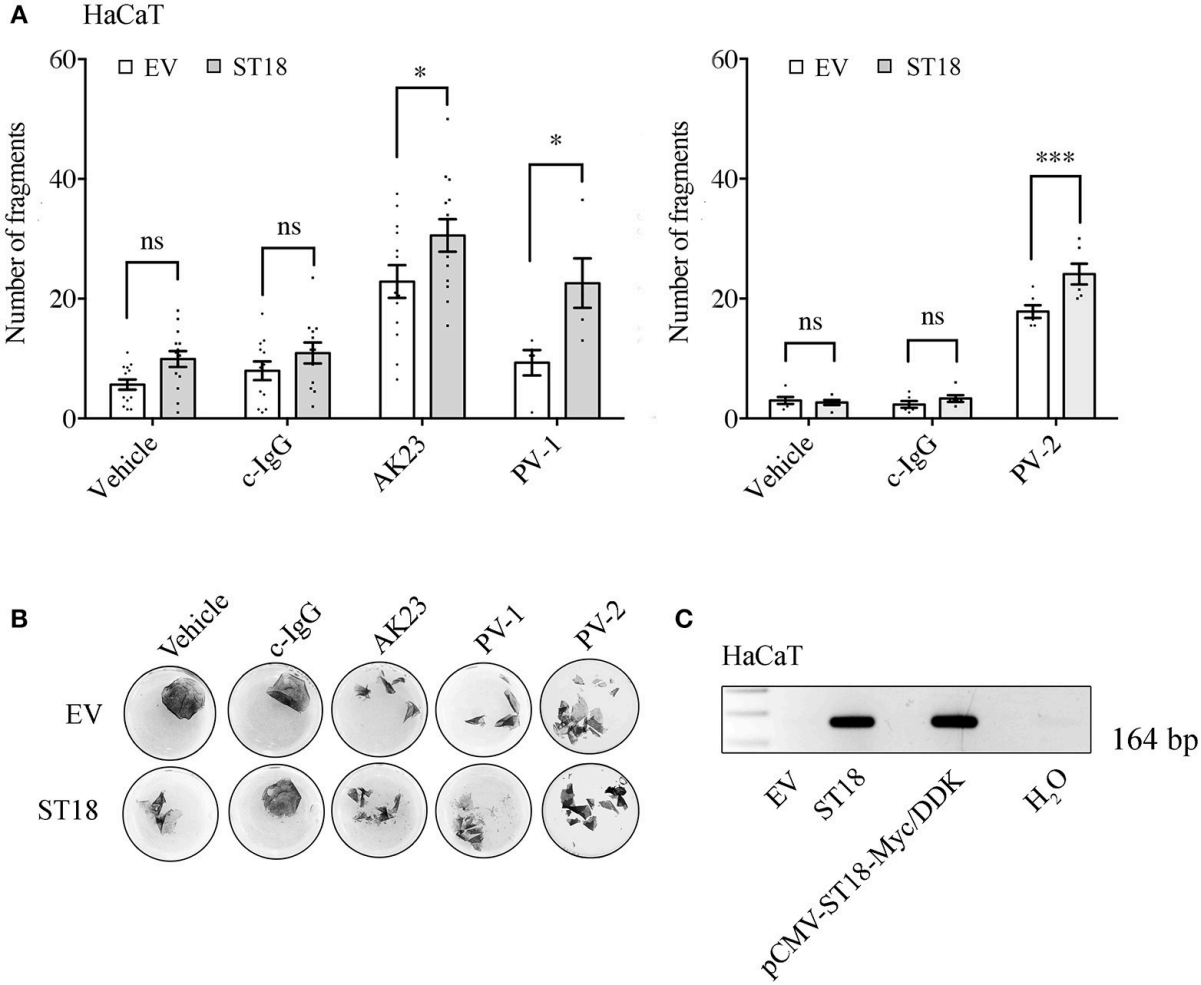
ST18 enhances AK23- and PV-IgG-mediated loss of cohesion in cultured keratinocyte monolayers. **(A)** Dispase-based dissociation assay in HaCaT cells transiently transfected with a control empty (EV) or a ST18-expression vector and exposed to either c-IgG, AK23, or PV-IgG fractions (*n* ≥ 0.05, two-way ANOVA, *p ≤ 0.05 and ***p ≤ 0.001 vs. respective monolayers transfected with EV). **(B)** Representative images of keratinocyte monolayers pre-stained with 10 μM MTT for better visualization of the fragments. **(C)** PCR analysis validating sufficient transfection efficiency was performed for each single experiment. Positive (pCMV-ST18-Myc/DDK vector) and negative (H_2_O) controls were run in parallel.

Recently, it has been reported that a PV-associated SNP leads to a significant increase in the expression of ST18 gene. As a consequence, in NHEK cells PV-IgG caused loss of keratinocyte adhesion as well as the release of key inflammatory molecules (29). In order to validate the effect of ST18 overexpression in human HaCaT keratinocytes, cells were transiently transfected with either empty vector (EV) or with ST18 expression vector. Consequently, the intact monolayers were exposed to control or pemphigus autoantibodies. Twenty four hours later, dispase-based dissociation assay was performed. Upon ST18 overexpression, the fragmentation of cell monolayers increased significantly in response to PV-IgG and AK23 incubation (Figures 2A,B). For each single experiment, sufficient transfection efficiency was confirmed by PCR analysis (Figure 2C).

### Effect of Pemphigus Autoantibodies and ST18 Overexpression on Cytokine Release

Besides preserving the structure of the skin by forming strong intercellular junctions and thus functioning as a physical barrier, keratinocytes also mediate inflammation by synthesizing and secreting various cytokines in response to physical or chemical damage, inflammatory signals or UV radiation (36–38). Therefore, in the present study, the pattern of cytokines expressed by non-transfected or transiently transfected human keratinocytes exposed to either c-IgG or pathogenic IgG was further analyzed.

**Figure 3:**
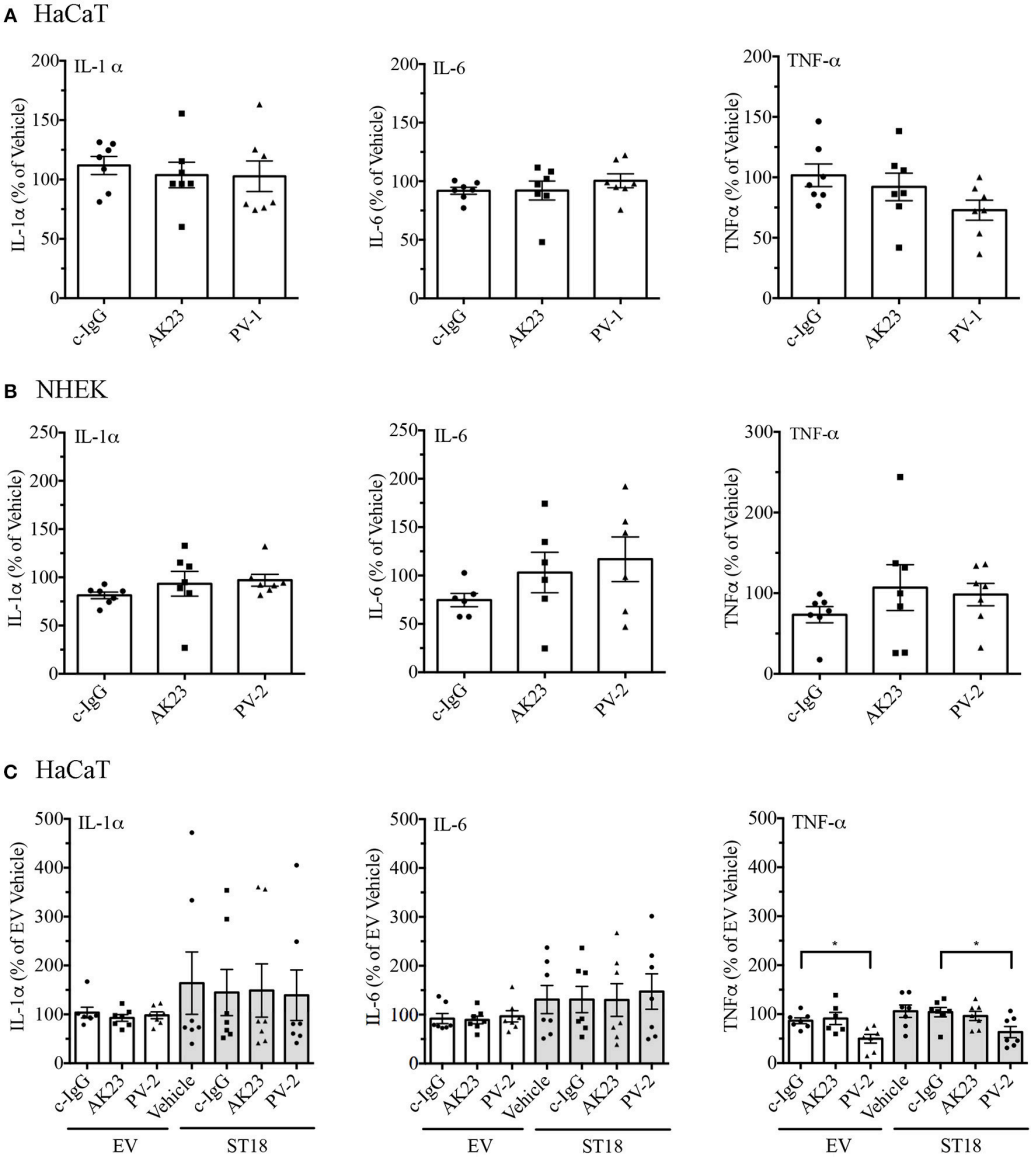
Release of pro-inflammatory cytokines in response to pemphigus autoantibodies and ST18 overexpression. The content of cytokines in supernatants collected from cell monolayers, which were either treated with controls (Vehicle, c-IgG), AK23 or PV-IgGs, was evaluated in both HaCaT **(A)** and NHEK **(B)** cells. **(C)** Cytokine release by HaCaT cells transiently transfected with either EV or ST18 expression vectors and exposed to PV-IgG or AK23 was determined. All data are presented as percent of Vehicle (*n* ≥ 6, two-way ANOVA; *p ≤ 0.05 vs. respective c-IgG).

Twenty four hours after reaching confluency HaCaT and NHEK cells were exposed to pathogenic IgG for another 24 h. Respective controls (PBS or c-IgG treated) were run in parallel. After the incubation was completed, supernatants were collected and simultaneously assessed for multiple cytokines by Luminex assay. Cytokine secretion of IL-1α, IL-6, TNF-α, and IFN-γ in both HaCaT and primary NHEK cells was not affected significantly (Figures 3A,B). In case of IFN-γ, independent on the treatment, cytokine release in HaCaT was either not altered, similar as in NHEK cells, or often under the detection limit (Supplementary Figures 2A,B). The latter observation was in line with a recent study in PV-patients, where the serum levels of IFN-γ were undetectable (39). Taken together, these data demonstrate that at least *in vitro*, cytokine release is not strictly required for loss of keratinocyte cohesion.

It was reported that ST18 overexpression in NHEKs results in a PV-IgG-mediated release of key inflammatory cytokines (29). In order to validate the effect of ST18 overexpression in human HaCaT keratinocytes, cells were transiently transfected with either EV or ST18- expression vector. For the recent study, experiments were carried out in HaCaT keratinocytes since the transfection efficiency in NHEK was not consistent. The intact monolayers were consequently exposed to IgG fractions. Supernatants were collected and secretion profiles of IL-1α, IL-6, TNF-α, and IFN-γ were analyzed by Luminex assay. ST18 overexpression induced no significant increase in the secretion of any cytokine analyzed (Figure 3C). However, IL-1α and IL-6 levels were augmented in some experiments, but not in others. When compared to c-IgG treatment, the release of TNF-α was even reduced significantly upon PV-IgG application in both EV and ST18 transfected monolayers. Secretion of IFN-γ was either undetectable or not affected by ST18 overexpression (Supplementary Figure 2C).

### Modulation of ERK Signaling Upon Treatment With PV-IgG Fractions and ST18 Overexpression

**Figure 4:**
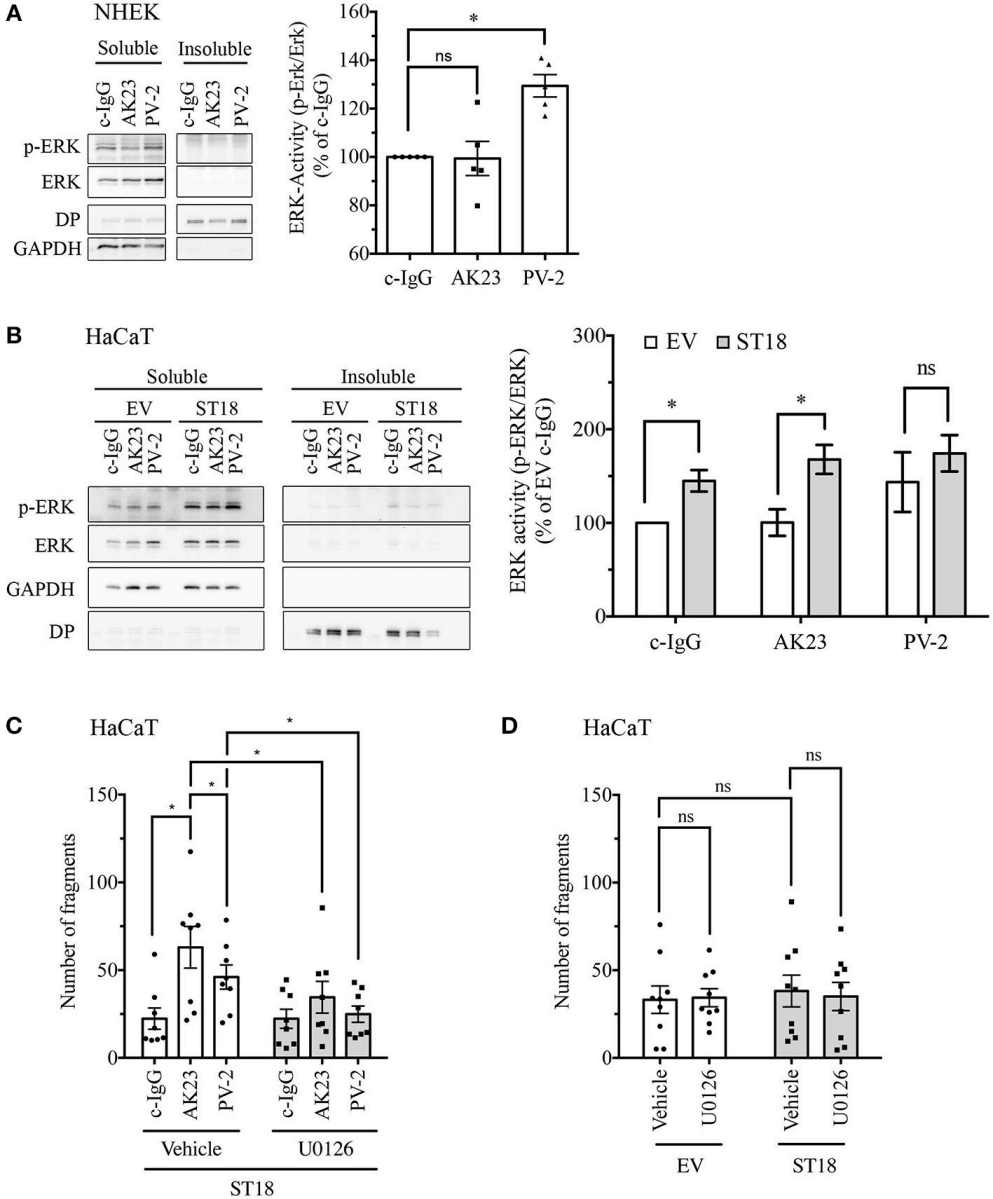
Modulation of ERK signaling after incubation with pemphigus autoantibodies and ST18 overexpression. Twenty four hours after differentiation, NHEK cells were exposed for 30 min to different IgG fractions. Triton X-100-soluble (cytosolic) and Triton X-100-insoluble (representing cytoskeletal- bound) fractions were generated and further analyzed by Western blot. **(A)** Representative blot of ERK phosphorylation in response to IgG for each Triton X-100 fraction produced. GAPDH was used as a cytosolic marker, while Desmoplakin (DP) served as a cytoskeletal-bound marker. Densitometric quantification of ERK activity (*n* = 5, One-Way ANOVA, *p < 0.05 vs. c-IgG). **(B)** Triton X-100 fractionation of HaCaT cells transfected with EV or ST18 vector and treated with IgG fractions for 30 min. Representative western blots showing total and phosphorylated ERK with respective markers for protein fractionation. Densitometric analysis of ERK activity within the soluble fraction using ImageJ software (*n* = 7, multiple *T*-tests, *p < 0.05). **(C)** In HaCaT cells, 24 h after ST18 transfection an U0126 was applied for an hour followed by 2 h incubation with different IgG fractions, as indicated. Dispase-based assay evaluating the effect of ST18 mediated ERK activation in cell monolayers treated with autoantibody fractions. **(D)** Impact of U0126-mediated ERK inhibition on baseline cell adhesion in EV- and ST18-transfected monolayers assessed by dispase-based dissociation assays (*n* ≥ 8, two-way ANOVA, *p < 0.05).

Several signaling pathways, including ERK signaling, have been implicated in the pathogenesis of pemphigus (16, 17). In order to evaluate ERK activity upon pemphigus autoantibody treatment, 24 h after differentiation with 1.8 mM Ca^2+^, confluent NHEK monolayers were incubated with different control or pathogenic IgGs for 30 min. ERK phosphorylation was investigated by triton fractionation and subsequent western blot analysis. The bands of phosphorylated and total ERK were densitometrically quantified and ERK activity was expressed as a ratio of phosphorylated-ERK to non-phosphorylated total ERK. Analysis revealed that compared to c-IgG, treatment with PV-2 IgG but not with AK23 induced significant activation of ERK (Figure 4A).

To evaluate the effect of ST18 overexpression on ERK signaling, 24 h after transfection HaCaT cells were incubated for 30 min with different IgGs (Figure 4B). Overexpression of ST18 resulted in significantly increased activity of ERK after c-IgG and AK23 treatment. Prominent but not significant elevation of ERK activity was observed in response to PV-2 application. Thus, we hypothesize that activation of ERK signaling is required to induce significant loss of cell cohesion as a result of ST18 overexpression. In order to test this hypothesis, inhibition of ERK activation by U0126, a widely used MEK inhibitor (40, 41), was performed in dispase assays using cell monolayers transiently transfected with either EV or ST18. Twenty four hours after transfection HaCaT cells were treated with 5 μM U0126 for an hour followed by incubation with either c-IgG, PV-IgG, or AK23 for another 2 h. Inhibition of ERK activation completely abolished autoantibody-induced loss of cell adhesion in ST18 transfected monolayers (Figure 4C).

Next, to test whether overexpression of ST18 modulates baseline adhesion via ERK, dispase-based dissociation assays were performed in vehicle or U0126-treated HaCaT keratinocytes transiently transfected with either EV or ST18 expression vector. Analysis revealed that neither in EV- nor in ST18- transfected cells adhesion was affected by ERK inhibition (Figure 4D). This indicates that an additional factor such as PV-IgG is required to facilitate ST18/ERK-mediated loss of adhesion.

## Discussions

Autoantibodies targeting Dsg1 and Dsg3 are pathogenic and cause blister formation by inducing structural desmosomal changes in the skin of PV patients (42). However, the mechanisms underlying disease development and the factors enhancing its manifestation have not yet been fully elucidated. Secondary factors promoting severity of the disease include non-Dsg antibodies (43) as well as genetic alterations such as recently reported ST18 SNPs (29–31) and may also entail keratinocyte-derived cytokine release.

Here, we investigate the effect of ST18 overexpression and cytokine secretion on PV-IgG mediated loss of adhesion. We observed that in both HaCaT and NHEK the release of key pro-inflammatory molecules such as IL-1α, IL-6, TNF-α, and IFN-γ is not strictly required for PV-IgG-induced loss of cell cohesion. However, ST18 overexpression did not only modulate cytokine release in some of the experiments but also altered ERK activity.

Pemphigus, as an autoimmune disease, depends highly on the activity of T-cells, the differentiation of which is regulated by autocrine cytokine signaling and is modulated by paracrine cytokine secretion from other cell type such as keratinocytes (44, 45). In skin, the production and secretion of cytokines has been recognized long ago and therefore intensely studied (46). These pro-inflammatory cytokines can also modulate keratinocyte behavior and could therefore be relevant for blister formation in pemphigus (47, 48).

Most studies on PV and cytokine release, however, are conflicting and report patient serum cytokine levels, which are most likely derived from T-cell signaling (49–52). Only few studies include cytokine assessment of blister fluid and only single reports evaluate production and secretion of cytokines *in vitro* (53). In the latter, polyclonal IgG fractions containing a plethora of antibodies, which could also include non-Dsg antibodies, were used (53). Therefore, in the present study, in addition to patient-derived PV-IgG the ability of a specific aDsg3 antibody, i.e., AK23, to induce cytokine secretion in keratinocytes was investigated. Both, AK23 and PV-IgG did not provoke significant cytokine secretion in HaCaT and NHEK cells (Figures 3A,B) but were efficient to impair keratinocyte adhesion. Therefore, secretion of keratinocyte-derived cytokines seems not to be primarily important for induction of loss of cell adhesion. Other antibodies such as mitochondrial antibodies or soluble FAS-ligand might change cytokine secretion profiles and thereby augment the disease as a secondary factor (54, 55).

Studies available on the transcription factor ST18 are limited. However, one of the first reports validated ST18 as a suppressor of tumor growth in breast cancer cell lines (56). Additionally, in fibroblasts, ST18 was reported to promote the expression of pro-inflammatory and pro-apoptotic genes, including cytokines (26). Furthermore, a relationship between a ST18 polymorphism and PV has now been shown to be relevant for Israelian, Egyptian (30) as well as Iranian (31) but not German (30) or Chinese (57) populations. In this respect, it was reported that increased loss of adhesion was associated with cytokine secretion in primary NHEK cells after introduction of ST18 expression vector and incubation with PV-IgG (29). Similarly, in HaCaT cells, we also detected decrease of cell adhesion upon simultaneous ST18 overexpression and pathogenic autoantibody treatment. However, we found elevated cytokine secretion in some but not all experiments following ST18 overexpression independently of IgG treatment. The discrepancy between both studies could possibly be based on the usage of different cell lines since in the study of Vodo et al. (29) NHEK cells were used whereas our experiments were performed with HaCaT cells. Nevertheless, these results indicate that mechanisms independent of cytokine secretion may also contribute to ST18-induced enhancement of adhesion loss.

Activation of several signaling pathways regulates desmosome stability and is involved in pemphigus skin blistering (58, 59). We focused on ERK because it was observed previously that this pathway is activated only when antibodies against Dsg1, which are known to be primarily important for epidermal blistering in pemphigus, were present in autoantibody fractions (1, 16, 17, 60). Similarly to ST18, ERK modulation also was shown to be associated with cytokine signaling (61–65) and could thereby link desmosome destabilization and ST18 expression. Here we show, that expression of ST18 induced significant ERK activation after c-IgG and AK23 treatment, which led to the hypothesis that ST18 promoted increase in disease severity is mediated by ERK activation independent of IgG treatment. In order to validate this hypothesis, ERK activation was inhibited by U0126. The mediator abolished loss of cell cohesion in monolayers transfected with ST18 and incubated with pemphigus antibodies indicating that ST18-mediated loss of adhesion is at least in part induced by ERK signaling pathway modulation.

Mechanisms underlying loss of cell adhesion in response to AK23 or PV-IgG may differ as the latter contain antibodies against Dsg1 and possibly against various other antigens (43). Moreover, it has been shown that in contrast to antibodies targeting Dsg3 such as AK23, which are capable to prevent homophilic interactions between Dsg3 molecules (66), antibodies targeting Dsg1 were not observed to inhibit Dsg1 interactions (34).

We previously reported that ERK was not activated by AK23 or mucosal-dominant PV-IgG both of which are targeting Dsg3, but rather by IgG fractions containing aDsg1 antibodies (17). Here we confirmed these results as we detected increased ERK phosphorylation after incubation with mucocutaneous PV-IgG but not AK23 in NHEK cells. However, the underlying ERK-dependent mechanisms relevant for pemphigus skin blistering are not yet completely understood. Interestingly, upon ST18 overexpression in HaCaT monolayers, ERK was activated under AK23 as well as c-IgG treatment, suggesting that ST18 may generally modulate ERK signaling. Nevertheless, further analysis revealed that baseline adhesion in ST18-transfected cells was unaffected by ERK inhibition (Figure 4D) indicating that PV-IgG is required as an additional factor to facilitate ERK-mediated loss of adhesion in response to ST18 overexpression.

Taken together, our study adds new insight on the mechanisms by which ST18 may contribute to pemphigus pathogenesis and indicates that altered signaling mechanisms regulating desmosomal adhesion may render keratinocytes more susceptible to autoantibody-induced loss of cell adhesion.

## Ethics Statement

Human sera were collected and used in accordance with recommendations of the ethics committee of the Philipps-Universität Marburg, Marburg, Germany. Name of the indicated project: Phänotypische und funktionelle Analyse von Immunzellen des peripheren Bluts beim Pemphigus vulgaris.

## Author Contributions

MR and EW performed the experiments, participated in study design, interpreted results, and wrote the manuscript. RS isolated the primary NHEK cells. JW designed the experiments, interpreted results, revised the manuscript and provided the funding. AY, NS, OS, and ES revised the manuscript and actively participate in the scientific discussions.

### Conflict of Interest Statement

The authors declare that the research was conducted in the absence of any commercial or financial relationships that could be construed as a potential conflict of interest.

## Supplementary Material

Supplementary Figure 1 NHEK monolayers treated with c-IgG, AK23 and PV-IgG were immunostained for Dsg3. Representative immunofluorescence images from n ≥ 4. Bar scale is 20 μm.

Supplementary Figure 2 Release of IFN-γ is unaffected by pemphigus autoantibody application or ST18 overexpression. Confluent keratinocyte monolayers were exposed to PV-IgG or AK23 for 24 h. Respective controls were run in parallel. IFN-γ concentration was assessed in HaCaT **(A)** and NHEK **(B)** cells. **(C)** HaCaT cells transiently transfected with control (EV) or ST18 expression vectors were treated as indicated. 24 h later, the release of IFN-γ was evaluated. Secretion of IFN-γ is presented as raw data (pg/ml), as each data point represents the mean of a single experiment. A dotted red line depicts the detection limit of the cytokine assay; *n* ≥ 6.
