# Supplementary figures and images for: ST18 Enhances PV-IgG-Induced Loss of Keratinocyte Cohesion in Parallel to Increased ERK Activation
Source: Front Immunol. 2019 Apr 17;10:770. doi: 10.3389/fimmu.2019.00770 (PMC6478701; doi:10.3389/fimmu.2019.00770)

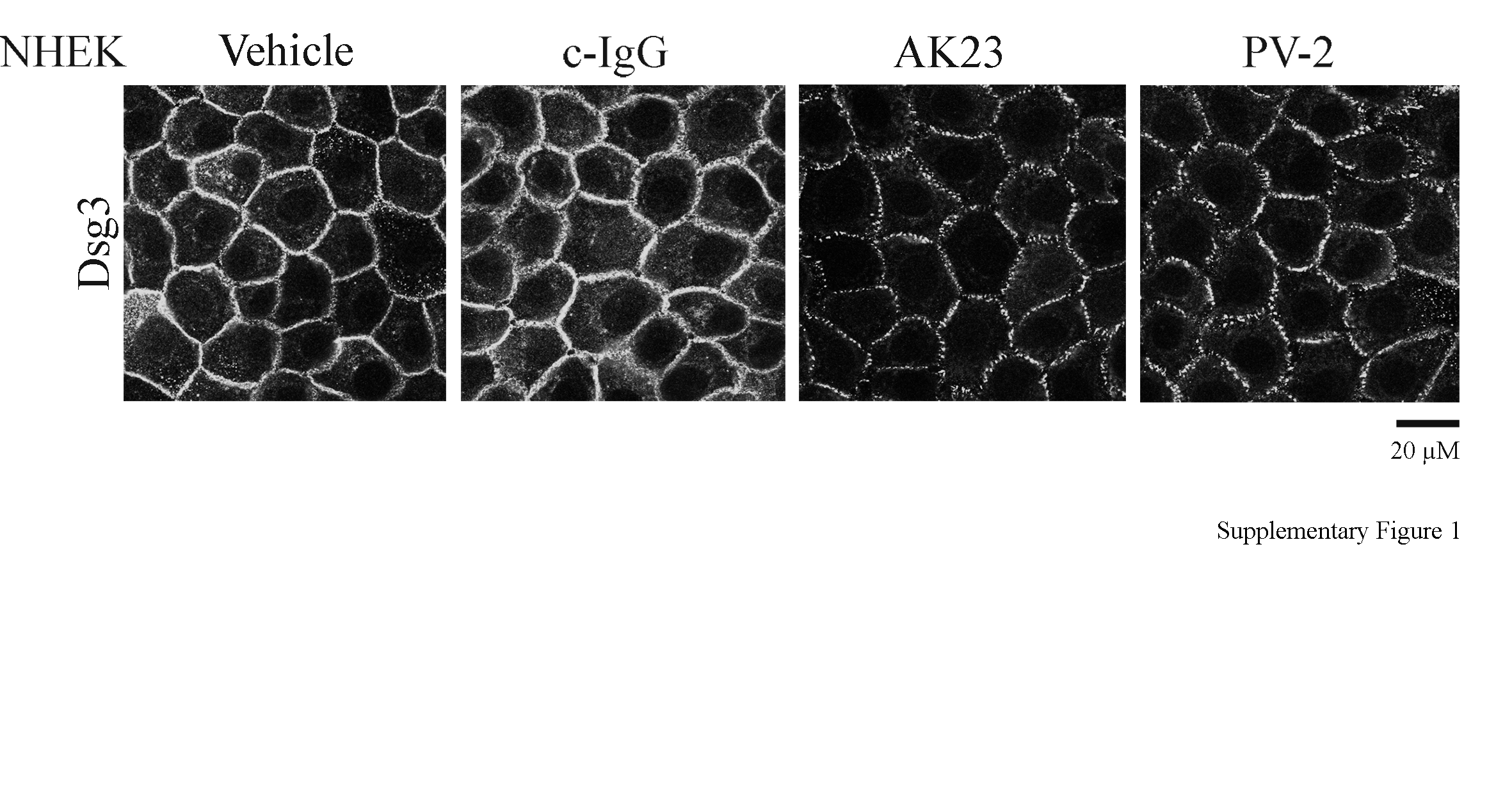

Supplement: Supplementary Figure 1 — NHEK monolayers treated with c-IgG, AK23 and PV-IgG were immunostained for Dsg3. Representative immunofluorescence images from n ≥ 4. Bar scale is 20 μm. [file Image_1.TIF]

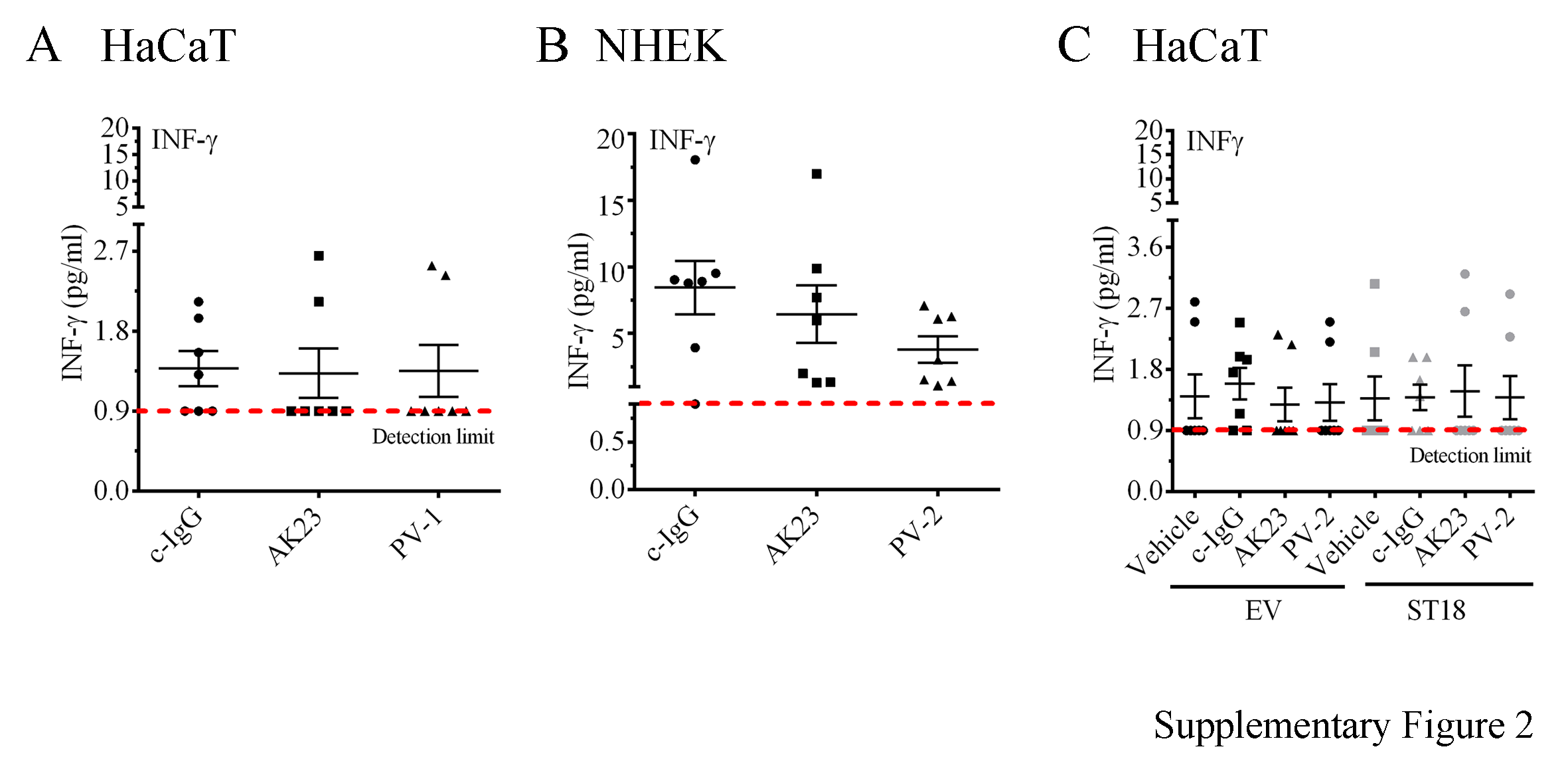

Supplement: Supplementary Figure 2 — Release of IFN-γ is unaffected by pemphigus autoantibody application or ST18 overexpression. Confluent keratinocyte monolayers were exposed to PV-IgG or AK23 for 24 h. Respective controls were run in parallel. IFN-γ concentration was assessed in HaCaT (A) and NHEK (B) cells. (C) HaCaT cells transiently transfected with control (EV) or ST18 expression vectors were treated as indicated. 24 h later, the release of IFN-γ was evaluated. Secretion of IFN-γ is presented as raw data (pg/ml), as each data point represents the mean of a single experiment. A dotted red line depicts the detection limit of the cytokine assay; n ≥ 6. [file Image_2.TIF]
